# Evolution of the Plasma and Tissue Kallikreins, and Their Alternative Splicing Isoforms

**DOI:** 10.1371/journal.pone.0068074

**Published:** 2013-07-10

**Authors:** Vassiliki Lila Koumandou, Andreas Scorilas

**Affiliations:** Department of Biochemistry and Molecular Biology, University of Athens, Athens, Greece; University of Patras, Greece

## Abstract

Kallikreins are secreted serine proteases with important roles in human physiology. Human plasma kallikrein, encoded by the KLKB1 gene on locus 4q34-35, functions in the blood coagulation pathway, and in regulating blood pressure. The human tissue kallikrein and kallikrein-related peptidases (KLKs) have diverse expression patterns and physiological roles, including cancer-related processes such as cell growth regulation, angiogenesis, invasion, and metastasis. Prostate-specific antigen (PSA), the product of the *KLK3* gene, is the most widely used biomarker in clinical practice today. A total of 15 KLKs are encoded by the largest contiguous cluster of protease genes in the human genome (19q13.3-13.4), which makes them ideal for evolutionary analysis of gene duplication events. Previous studies on the evolution of KLKs have traced mammalian homologs as well as a probable early origin of the family in aves, amphibia and reptilia. The aim of this study was to address the evolutionary and functional relationships between tissue KLKs and plasma kallikrein, and to examine the evolution of alternative splicing isoforms. Sequences of plasma and tissue kallikreins and their alternative transcripts were collected from the NCBI and Ensembl databases, and comprehensive phylogenetic analysis was performed by Bayesian as well as maximum likelihood methods. Plasma and tissue kallikreins exhibit high sequence similarity in the trypsin domain (>50%). Phylogenetic analysis indicates an early divergence of KLKB1, which groups closely with plasminogen, chymotrypsin, and complement factor D (CFD), in a monophyletic group distinct from trypsin and the tissue KLKs. Reconstruction of the earliest events leading to the diversification of the tissue KLKs is not well resolved, indicating rapid expansion in mammals. Alternative transcripts of each *KLK* gene show species-specific divergence, while examination of sequence conservation indicates that many annotated human KLK isoforms are missing the catalytic triad that is crucial for protease activity.

## Introduction

Peptidases or proteases, are enzymes that break down a polypeptide or protein by cleaving peptide bonds. Proteolytic enzymes, their substrates and inhibitors, are of great interest in biology, medicine, and biotechnology. Apart from their role in the breakdown of misfolded or unnecessary proteins, proteases affect the localization and activity of many gene products, and are of great importance to signaling pathways. Their function thus affects cell proliferation and differentiation, immunity, homeostasis, inflammation, blood coagulation, autophagy, necrosis, and apoptosis [1]. Alterations or malfunction of proteolytic systems underlie multiple pathological conditions such as cancer, inflammatory and cardiovascular diseases. Therefore, many proteases are intensely studied as potential drug targets or as diagnostic and prognostic biomarkers [2]. Based on their structure, proteases have been classified into homologous families, one of the biggest of which are the serine proteases (named for the nucleophilic Ser residue in their active site), which includes trypsin and chymotrypsin [1], [3], [4]. Plasma and tissue kallikreins are members of the serine protease family S1 (S1A subfamily of the PA(S) serine peptidase clan [4], [5]).

Kallikreins are secreted serine proteases that are synthesized as pre-proenzymes, the signal peptide of which is cleaved for secretion; the proenzyme is also cleaved for full activation [6]. Human plasma kallikrein (PK), encoded by the KLKB1 gene on human chromosome 4q34-35, is synthesized in the liver as an inactive precursor and circulates in the plasma. Once activated by coagulation factor XII, plasma kallikrein liberates kinins (bradykinin and kallidin) from high molecular weight kininogen (HK). The kinins mediate blood coagulation, activation of inflammation, regulation of blood pressure, and modulation of thrombosis risk independently of homeostasis, via vasodilation. Activated coagulation factor XII (FXII) and PK can cleave and activate each other in a positive feedback loop. The plasma kallikrein-kinin system can be activated either by contact activation (when blood or plasma interacts with artificial surfaces) leading to coagulation, or, under physiological conditions, by PK and HK binding to endothelial cells [7], [8]. Patients with FXII, PK and HK deficiency are exceedingly rare, and thus too few to characterize a common clinical phenotype [8].

Human tissue kallikrein (KLK1) was first described in the 1930s [9], and named after the organ where it was observed, the pancreas (“kallikreas” in Greek). It has similar activity to plasma kallikrein, but cleaves low molecular weight kininogen (LK) to release lysyl-bradykinin (Lys-BK), which mediates regulation of blood pressure, smooth muscle contraction, vascular permeability, vascular cell growth, and inflammatory cascades, electrolyte balance, neutrophil chemotaxis, and pain induction [10]. A further two tissue kallikreins (KLK2 and KLK3) were discovered in the 1980s [11], [12], [13], [14]. KLK3 is also known as prostate cancer antigen (PSA) and is widely used as a diagnostic biomarker for prostate cancer [15]. Apart from these three “classical” KLKs, the tissue kallikrein family is now known to be composed of 15 members in total, after the discovery of the kallikrein-related peptidases KLK4-15 [16], [17]. Human tissue kallikreins share significant sequence (>40%) and structural similarity, as well as conserved exon/intron structure, but only KLK1 has been reported to have significant kininogenase activity [6], [10], [18]. Based on structural comparisons, the substrate specificity of kallikreins is probably defined by residues in the loops surrounding the mouth of the active site and by charged surface regions that can act as exosites for substrate recognition [19], [20] The expression of KLKs is regulated by steroid hormones, resulting in diverse expression patterns and functions, including roles in tissue remodeling, prohormone processing, neural plasticity, as well as cancer-related processes such as cell growth regulation, angiogenesis, invasion, and metastasis [6], [18], [20]. For example, KLK4 has a role in tooth development; KLK5, KLK7 and KLK14 are involved in skin desquamation; KLK6, KLK8 and KLK11 are involved in neural plasticity; KLK2 and KLK3 are involved in reproduction (liquefaction of semen); KLK1, KLK2, KLK4 and KLK6 are involved in ECM remodeling and tumor invasion [6], [18], [20]. Aberrant expression of distinct KLKs is associated with clinicopathological parameters; therefore, KLKs are intensely studied for their potential as cancer biomarkers, although no such role has been assigned to plasma kallikrein so far.

Interestingly, all 15 KLKs are encoded by the largest contiguous cluster of protease genes in the human genome, located in the genetic locus 19q13-14 [21]. This makes the family an ideal candidate for studying the evolution of gene duplication events leading to this diversification [22]. Indeed, comparative genomics analysis has resulted in the identification of orthologs in most mammals and marsupials for which complete genome information is available, while the contiguous gene organization of this locus is also largely conserved [22], [23]. Phylogenetic analysis of the tissue kallikreins has illustrated that the classical tissue kallikreins KLK1, KLK2, and KLK3 form a monophyletic group where KLK2 and KLK3 seem to be recent derivatives [22], [23]; in agreement with this, KLK3 is only found in the primates [22], [24], [25]. Bayesian analysis of the human, chimpanzee, mouse, rat, dog, pig, and opossum kallikreins indicated monophyletic groups (subfamilies) for KLK8 and the classical kallikreins (KLK1, KLK2, and KLK3); KLK4 and KLK5 (including possibly also KLK14); KLK10 and KLK12; and KLK9, KLK11, and KLK15, which was considered basal as it grouped closest to trypsin, used as an outgroup for the family [23]. More recently, kallikrein homologs were identified in evolutionarily distant species (frog, lizard, turkey and Zebra Finch), indicating an early origin of kallikreins at ∼330 mya [22]. Maximum likelihood phylogenetic analysis provided further evidence for the subfamilies consisting of the classical kallikrein subfamily (KLK1, KLK2, and KLK3); KLK4 and KLK5; KLK10 and KLK12; and KLK9, KLK11. KLK15 as well as the non-eutherian kallikreins appear basal to the group with trypsin as an outgroup [22].

In the present study we investigate the phylogenetic relationship of plasma and tissue kallikreins, which has not been studied so far. We also re-examine the phylogenetic position of the early diverging kallikreins from non-mammals. Furthermore, we examine the evolution of protein-coding alternative transcripts of the kallikrein family, particularly in humans, as well as the conservation of critical functional residues [5], required for protease activity, in these kallikrein isoforms. Alternative splicing contributes to the diversification of the transcriptome and proteome, especially in metazoa and in humans [26], [27]. Multiple alternative transcripts have been reported for most human KLKs, and many encode different protein isoforms, some of which appear to be specifically expressed in cancer tissues [28], [29]. The relatively recent origin of the tissue kallikrein family, and its expansion via gene duplications, makes it an interesting candidate for studying the evolution of alternative splicing, both among members of this gene family, and between species. Therefore, examining the evolution of the kallikrein protein isoforms resulting from alternative splicing can answer the question of whether these have evolved (a) in a kallikrein-type-specific manner, and (b) in a species-specific manner.

## Methods

### Database Sequence Search

All annotated plasma kallikrein and tissue kallikrein sequences, as well as representative serine proteases (trypsin, chymotrypsin, plasminogen, and complement factor D), were collected from NCBI (RefSeq) and/or from the Ensembl database (release 66) for the following species: *Homo sapiens* (human), *Mus musculus* (mouse), *Bos taurus* (cattle), *Canis familiaris* (dog), *Monodelpis domestica* (opossum), *Procavia capensis* (hyrax), *Macropus eugenii* (wallaby), *Xenopus tropicalis* (frog), *Ornithorhynchus anatinus* (platypus), *Anolis carolinensis* (lizard), *Meleagris gallopavo* (turkey), *Taeniopygia guttata* (Zebra Finch), and *Gallus gallus* (chicken). The sequences of all annotated protein-coding alternative transcripts were also collected, and are presented in Table S1, along with the correspondence between the RefSeq and Ensembl sequences.

### Genetic Locus Analysis

In most mammals, tissue kallikreins are encoded by adjacent genes [22]. Therefore, for the earlier diverging species, where some kallikreins are reportedly missing, the dataset of annotated sequences was supplemented with non-annotated kallikrein-like proteins, encoded by genes adjacent to the ones already identified, by examining the genetic locus neighborhood of all identified sequences on the NCBI Gene and Ensembl databases.

### Identification of Functional Domains, Exon/intron Boundaries, and Different Types of Alternative Splicing Patterns

All sequences were parsed through the Conserved Domain Database (CDD) at NCBI [30], and the SMART tool [31], to identify conserved functional domains, as well as exon/intron boundaries and intron phases (SMART annotation of intron positions is based on Ensembl predictions). Tissue kallikrein isoforms were also classified based on the different types of alternative splicing used to generate structural diversity. These classifications (alternative promoter, exon skipping, alternative 3′ SS selection, alternative 5′ SS selection, alternative poly(A)) were as described previously [27].

### Alignments and Phylogenetic Analysis

Alignments were created using MUSCLE [32], and checked visually using Jalview [33]. Only unambiguous homologous regions were retained for phylogenetic analysis; manual masking and trimming was performed in MacClade [34]. Masked and trimmed alignments (Alignments S1, S2, S3, S4, S5, S6) are provided as supplementary simple text files (in Phylip3.6 format). One dataset included all sequences (KLKs-all) (Alignments S5 and S6); two reduced datasets was also analyzed: one contained only the longest alternative transcript for each sequence (KLKs-one) (Alignments S3 and S4), and the other contained all human protein-coding isoforms (KLKs-all_human) (Alignments S1 and S2). ProtTest [35] was used to estimate the appropriate model of sequence evolution. Phylogenetic analysis was performed by three separate methods. To obtain the Bayesian tree topology and posterior probability values, the program MrBayes version 3.1.2 was used [36]. Analyses were run for 1–3×10^6^ generations, removing all trees before a plateau established by graphical estimation. All calculations were checked for convergence and had a splits frequency of <0.1. Maximum-likelihood (ML) analysis was performed using PhyML [37] and RAxML [38] with 100 bootstrap replicates. Nodes with at least 0.95 posterior probability and 80% bootstrap support were considered robust, and nodes with at least 0.80 posterior probability and 50% bootstrap support are shown. The alignment of all the human protein-coding isoforms (Alignment S1) was also used to check for the presence/absence of the residues which constitute the catalytic triad, required for protease activity [5].

## Results and Discussion

### Domain Structure and Sequence Conservation Across the Human Kallikreins

**Figure 1:**
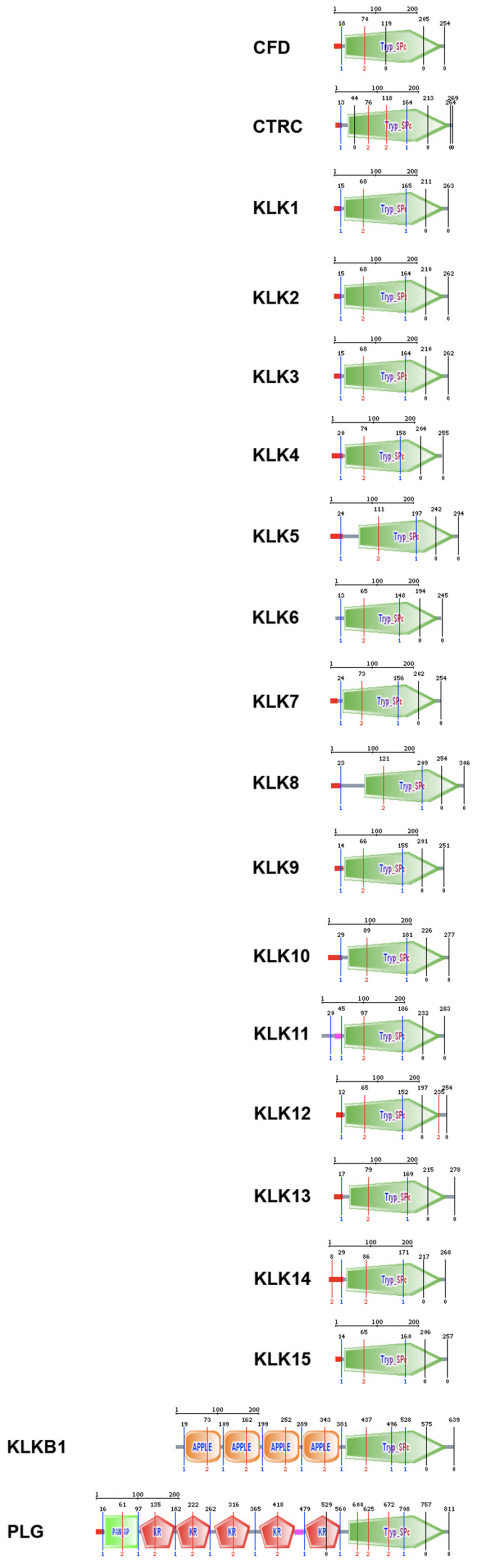
Conserved domains and exon boundaries identified via SMART for the human plasma kallikrein, tissue KLKs, and other serine proteases. All proteins contain a Trypsin-like serine protease domain (green; IPR001254), and most have an N-terminal signal peptide (red). Plasma kallikrein (KLKB1) contains four N-terminal APPLE domains (orange; IPR000177), also found in coagulation factor XII, and mediating protein-protein interactions. Plasminogen (PLG) contains an N-terminal APPLE domain, as well as five Kringle domains (red; IPR000001). Intron positions are indicated with vertical lines showing the intron phase (below) and exact amino acid position (above).

Human plasma and tissue kallikrein amino acid sequences were collected from the NCBI and Ensembl databases, including all annotated protein-coding alternative transcripts (Table S1). The human tissue KLK protein sequences have an average length of approximately 260 amino acids, and contain a trypsin-like protease domain that spans almost the full length of the protein (Figure 1). The human plasma kallikrein is 638 amino acids long and contains the trypsin-like protease domain in the C-terminus, as well as four APPLE-Factor-XI-like domains in the N-terminus (Figure 1). The trypsin-like protease domain is shared by a large family of serine proteases [4]. The PAN/APPLE domains mediate protein-protein or protein-carbohydrate interactions; these N-terminal domains in plasma kallikrein mediate binding to high-molecular-weight kininogen, and dimerization in Factor XI [39].

Although the sizes of the human tissue *KLK* genes range from 4 up to 10 kb, the five coding exons are highly conserved both in size and organization, so most of the differences relate to differences in intron sizes [6], [14], [20], [40]. The human *KLKB1* gene spans ∼30 kb and comprises 14 coding exons. The C-terminus of human plasma kallikrein, which contains the trypsin-like domain, is homologous to the tissue kallikreins, showing, on average, 36% identity and 52% similarity. When comparing the homologous region between plasma and tissue kallikreins, the exon/intron boundaries and intron phases are largely conserved (Figure 1). The only exception is that plasma kallikrein contains one extra intron, i.e. the corresponding third exon from tissue kallikreins is now split into two exons (Figure 1). This conservation of the intron organization is more striking between the human plasma and tissue kallikreins than when comparing kallikreins to other serine proteases, such as chymotrypsin, plasminogen, or complement factor D (Figure 1) suggesting a closer evolutionary relationship between human plasma and tissue kallikreins, than among serine proteases in general.

**Figure 2:**
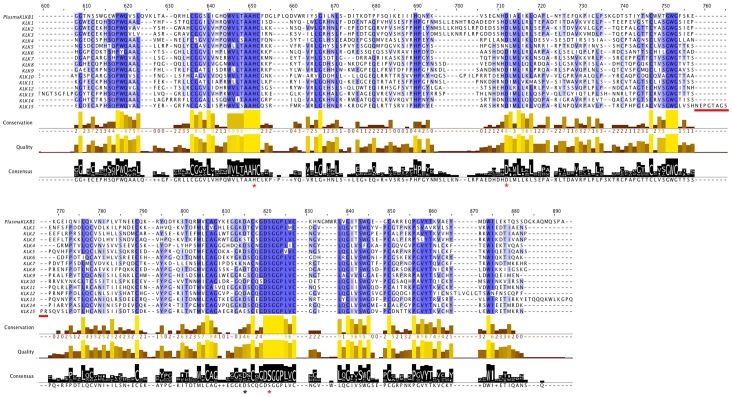
Multiple sequence alignment of human kallikreins. The longest alternative transcript for each of the human tissue kallikreins, as well as the plasma kallikrein, were aligned using Muscle (image generated with Jalview). Red asterisks indicate the catalytic triad residues (trypsin numbering: His41, Asp96, Ser189), and the black asterisk indicates the residue determining trypsin or chymotrypsin substrate cleavage specificity (Asp183). The 10-amino acid insertion in the 148 loop of KLK15 [20] is underlined in red. The amino acid position numbers shown at the top correspond to the full alignment, including the sequences for all the human alternative transcripts, given in Figure S1 (also see Alignment S1). Here, we focus on the part of the alignment corresponding to the homologous region between plasma and tissue kallikreins, encompassing the trypsin-like domain. Conserved residues are highlighted (blue: >80% agreement, mid blue: >60% agreement, light blue: >40% agreement; only the residues that agree with the consensus residue for each column are coloured). The conservation annotation histogram below the alignment reflects conservation of the physicochemical properties, and marks absolutely conserved residues (score 11) with a yellow asterisk ‘*’, and columns where physicochemical properties are conserved (score 10) with a yellow ‘+’; less conserved positions are shown in darker colours with decreasing score. The quality annotation histogram reflects the likelihood of observing a mutation in any particular column of the alignment based on the BLOSUM62 matrix scores (for each column, the sum of the ratios of the two BLOSUM62 scores for a mutation pair, and each residue’s conserved BLOSUM62 score, are normalised and plotted on a scale of 0 to 1) The consensus histogram reflects the percentage of the modal residue per column, and the consensus sequence logo is shown below the sequence for conserved regions (‘+’ denotes non-conserved residues and ‘−’ denotes gap residues).

**Figure 3:**
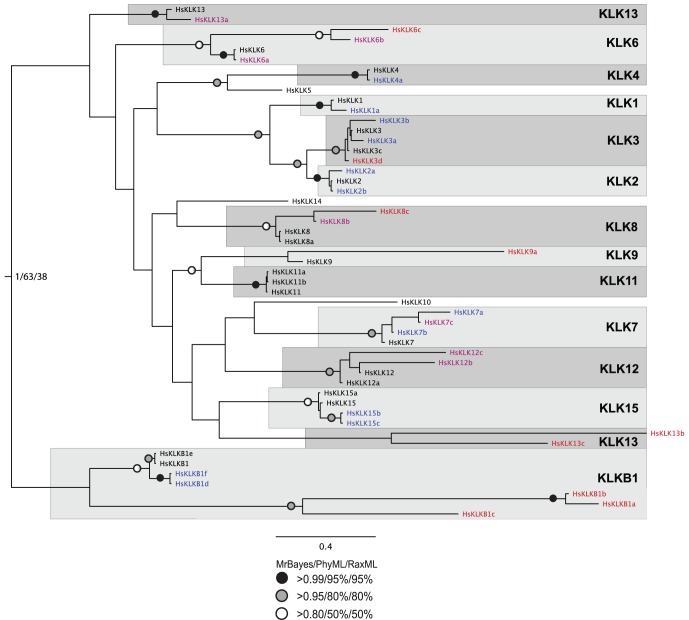
Phylogenetic reconstruction of human plasma and tissue kallikreins, including all annotated protein-coding alternative transcripts. The tree shown is the best Bayesian topology. Numerical values at the nodes of the tree (x/y/z) indicate statistical support by MrBayes, PhyML and RAxML (posterior probability, bootstrap and bootstrap, respectively). Values for highly supported nodes have been replaced by symbols, as indicated. Colored protein names indicate that, in the particular sequence, the crucial catalytic triad (trypsin numbering: His41, Asp96, Ser189) for protease activity is not conserved; specifically, blue indicates that one of the three residues is missing, purple indicates that two of the three residues are missing, and red indicates that all three residues are missing. Full details and accession numbers for all protein sequences used are given in Table S1; also see Alignments S1 and S2, for the masked and trimmed alignments used to construct the tree.

The classical tissue kallikreins (KLK1, KLK2, and KLK3) share 62–67% amino acid identity, while KLK4-15 share 27-39% identity among them, and exhibit 25-39% identity to KLK1 [18]. Multiple sequence alignment of the human tissue kallikreins with plasma kallikrein (Figure 2, Figure S1, Alignment S1) indicates good sequence conservation along the whole length of the trypsin-like domain. The catalytic triad that is critical for serine protease activity is encoded by His41, Asp96, and Ser189 (trypsin numbering; marked by red asterisks in Figure 2), located in the second, third, and fifth coding exons, respectively, and is highly conserved across all kallikreins (Asp/Ser183 is the recognition site in the substrate-binding pocket that determines the trypsin or chymotrypsin substrate cleavage specificity, respectively; marked by a black asterisk in Figure 2) [5], [18]. Many residues around the catalytic triad are also highly conserved (highlighted in blue in Figure 2). However, several protein-coding alternative transcripts are often missing whole exons. Based on the conservation of the catalytic triad, a prediction can be made about whether they have protease activity. Therefore, we checked if the catalytic triad was conserved in all human KLK isoforms based on the full-length alignment given in Figure S1 (also see Alignment S1). The results (presence or absence, scored with “Y” or “N”, respectively, shown in the last column of Table S1), indicate that many protein-coding alternative transcripts for human kallikreins are predicted to lack full protease activity, as highlighted in Figure 3.

### Phylogeny of Tissue and Plasma Kallikreins

Apart from human, KLK homologs in various organisms (mouse, cattle, dog, opossum, hyrax, wallaby, frog, platypus, lizard, turkey, Zebra Finch, and chicken) have been identified [22]. We supplemented these sequences with additional BLAST and domain searches, where needed. The species were chosen to cover the diversity of organisms studied, but exclude the most closely related species, as their sequences are bound to provide redundant information and are unlikely to provide extra resolution at the base of the tree. Amino acid sequences were collected from the NCBI and Ensembl databases, including all annotated protein-coding alternative transcripts for each member of the plasma and tissue kallikrein family, and are shown in Table S1, along with the correspondence between the RefSeq and Ensembl sequences. Overall, a good correspondence between annotation in RefSeq and Ensembl was seen, apart from the *Canis familiaris* genome, where some discrepancies in the annotation between the two databases are noted in Table S1.

**Figure 4:**
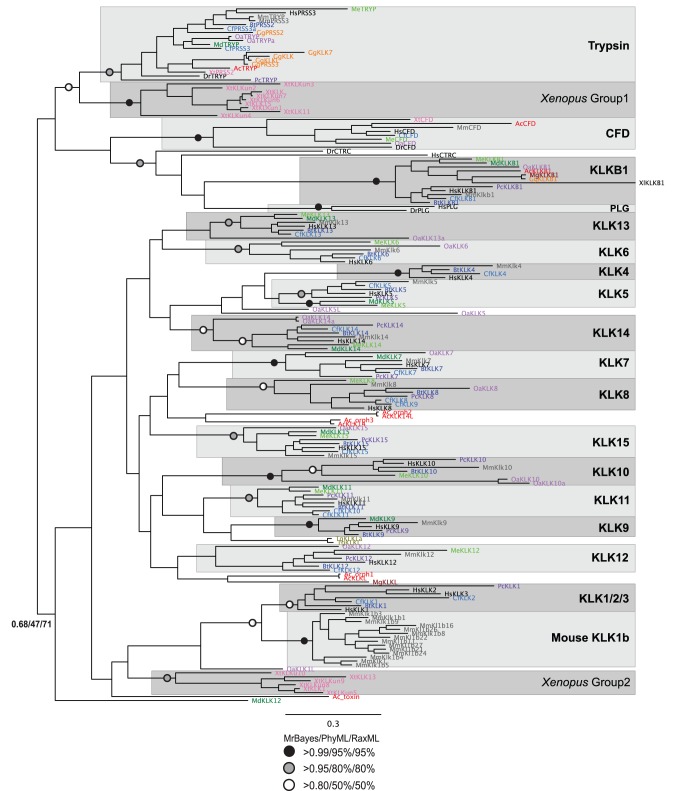
Phylogenetic reconstruction of plasma and tissue kallikreins in the context of other serine proteases, including trypsin, chymotrypsin, plasminogen, and complement factor D. The tree shown is the best Bayesian topology. Numerical values at the nodes of the tree (x/y/z) indicate statistical support by MrBayes, PhyML and RAxML (posterior probability, bootstrap and bootstrap, respectively). Values for highly supported nodes have been replaced by symbols, as indicated. Species names are abbreviated and colored as follows: Hs - *Homo sapiens* (human, black); Mm - *Mus musculus* (mouse, grey); Bt - *Bos taurus* (cattle, blue); Cf - *Canis familiaris* (dog, light blue); Md - *Monodelphis domestica* (opossum, green); Me - *Macropus eugenii* (wallaby, light green); Pc - *Procavia capensis* (hyrax, purple); Xt - *Xenopus tropicalis* (frog, pink); Oa - *Ornithorhynchus anatinus* (platypus, light purple); Ac - *Anolis carolinensis* (lizard, red); *Meleagris gallopavo* (turkey, brown); Gg - *Gallus gallus* (chicken, orange); Tg - *Taeniopygia guttata* (Zebra Finch, khaki); Dr - *Danio rerio* (zebrafish, black). Full details and accession numbers for all protein sequences used are given in Table S1; also see Alignments S3 and S4, for the masked and trimmed alignments used to construct the tree.

To examine the evolution of plasma and tissue kallikreins, phylogenetic analysis was based on two datasets, one including all alternative transcripts (KLKs-all), and the other including only the longest alternative transcript for each gene (KLKs-one). The analysis was performed using Bayesian and maximum likelihood methods, and nodes supported by high posterior probability and bootstrap values by all three methods were considered robust. The Bayesian topology for the KLKs-one dataset is shown in Figure 4. The trypsin clade is well supported and includes representatives from mammals, chicken, lizard, frog, and fish. Moreover, the plasma kallikrein (KLKB1) clade is well supported, as is its connection to chymotrypsin (CTRC) and plasminogen (PLG). The KLKB1 clade includes orthologs from chicken, frog and lizard, indicating an early origin for plasma kallikrein in aves and amphibia. Plasma kallikrein is not found in fish, although homologs of coagulation factor XI and the PAN_APPLE domain are found in certain fish species (e.g. PLG of *Danio rerio*). This, together with our results, indicates that plasma kallikrein originated sometime before the evolution of the tetrapoda, and thus the origins of the surface-dependent blood coagulation pathway, as it is known in mammals, can be traced to the ancestor of the amphibia.

The tissue kallikreins consistently group together, and separate from trypsin and KLKB1; however, the monophyly of the tissue KLKs is only marginally supported by bootstrap and posterior probability values (Figure 4). Mouse KLK1 groups with the other mouse KLK1b sequences, as a sister group to the KLK1/2/3 clade composed of human, cattle, dog, hyrax, and turkey KLK1, human and dog KLK2, and human KLK3. Clades for the other tissue kallikrein members group with good statistical support indicating clear monophylletic groups for each kallikrein type (black dots in the nodes for KLK4, KLK7, KLK9 and KLK10; grey dots in the nodes for KLK6, KLK11– except for the platypus KLK11– KLK13 and KLK15; white dots in the nodes for KLK8 and KLK14). The KLK5 clade has good support for two subclades, one including human, mouse, dog, cattle, and hyrax, and the other including opossum and wallaby. KLK12 members consistently group together (except for the opossum KLK12) but not with significant statistical support. Similar results for the connection between the classical KLKs1/2/3 and the mouse KLK1b clades, as well as the monophyly of each kallikrein subtype have also been reported in previous studies [22], [23]. Therefore, the expansion of the classical KLKs is considered a relatively recent event [22], [23]. However, the inter-relationships between the kallikrein members are not well resolved, so it is difficult to confidently infer the order of events leading to the expansion of the tissue kallikreins; this result indicates a rapid expansion of the tissue kallikrein family in mammals. It is nevertheless also possible that the signal of these events might have been lost due to sequence divergence, and to the relatively small size of kallikrein proteins.

The tight monophylletic groupings for each kallikrein type suggest that their distinct functions are conserved in different species. In support of this, variations in the site of the substrate-binding pocket that defines the trypsin or chymotrypsin substrate cleavage specificity of each kallikrein, are conserved between species. The substrate specificity of kallikreins depends on a residue which lies at the base of the substrate binding pocket; most kallikreins retain the aspartate at that position (Asp183, indicated by a black asterisk in Figure 2), which confers trypsin-like specificity to cleave after arginine or lysine residues [20]. However, human KLK7 has asparagine, which defines chymotrypsin activity [20], and the KLK7 orthologs in mouse, dog, cattle, hyrax and opossum also have asparagine at that position, which suggests that the chymotrypsin-like activity of KLK7 is conserved across multiple species (Alignment S3). Human KLK9 has glycine at that position, which indicates that it cleaves after valine and alanine residues [20]; the glycine at that position is conserved in the mouse, cow, hyrax and opossum KLK9 orthologs, but not in the annotated dog KLK9 (Alignment S3), which does not group with other KLK9s in the phylogenetic tree. Finally, human KLK15 has a glutamate at that position, which confers trypsin-like specificity [20]; the glutamate is also seen in dog KLK15, but most other KLK15s have aspartate at that position, while cow KLK15 has asparagine indicating chymotrypsin-like activity (Alignment S3). KLK substrate specificity is further defined by the sequence of eight surface-exposed loops surrounding the active site [19], [20]. For example, human KLK15 has a 10- amino acid insertion in the 148 loop [20] (underlined in red in Figure 2); a 10-amino acid insertion (with only partly conserved sequence) is also found at the same position in the mouse, dog, cattle, hyrax and opossum KLK15 orthologs, while wallaby has a 6-amino acid insertion (Alignment S3). This provides further evidence for conserved substrate specificity in KLK orthologs between species.

**Figure 5:**
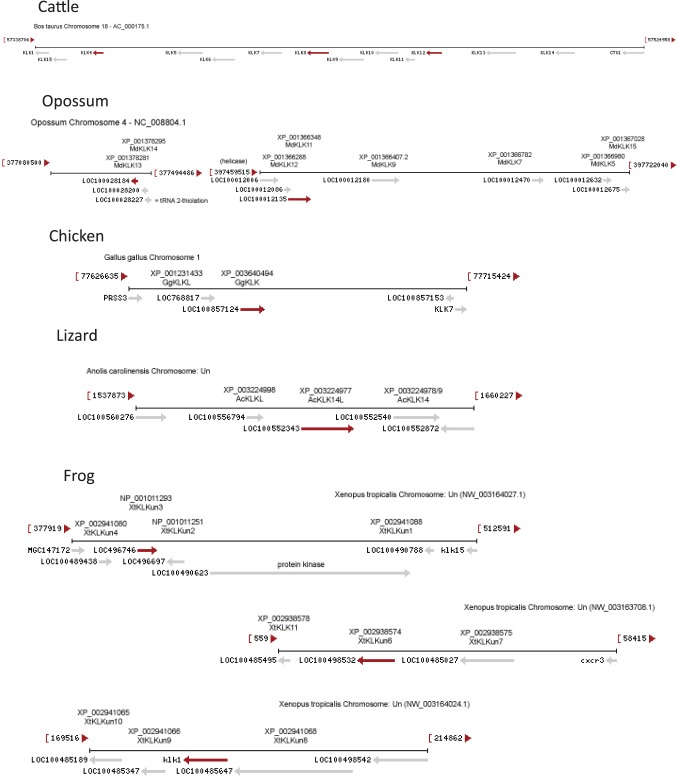
KLK gene clusters in selected species, as determined by examining the NCBI Gene database. The genetic locus neighborhood for *KLK*s in cattle, opossum, chicken, lizard, and frog are shown. The *KLK* gene cluster in cattle (*Bos taurus* chromosome 18) has a similar structure to the one in human, mouse and dog, except that it is missing the KLK2 and KLK3 genes (see Figure S2). The *KLK* gene cluster in the opossum (*Monodelphis domestica* chromosome 4) contains a smaller number of genes, but again has a similar structure, except that KLK13 and KLK14 are not adjacent to the other KLKs and that they are transcribed in the opposite direction, indicating a chromosomal rearrangement. In the chicken (*Gallus gallus* chromosome 1), three trypsin-like proteins adjacent to the annotated KLK7 were identified as putative novel KLKs (GgKLK, GgKLKL, GgPRSS3). Similarly, in the lizard (*Anolis carolinensis*) two putative novel KLKs (AcKLK14L, AcKLKL) were identified adjacent to the annotated KLK14. Finally, in the frog (*Xenopus tropicalis*) ten putative novel KLKs (XtKLKun1-10) were identified adjacent to annotated KLKs. Note that the lizard and frog sequences are on parts of the genome that are not fully assembled yet.

Given that tissue kallikreins are encoded in a contiguous gene cluster in mammals, the genetic locus neighborhood of all identified tissue kallikrein sequences in non-mammals was also checked on the NCBI Gene and Ensembl databases to look for non-annotated kallikrein-like proteins adjacent to the ones already identified. In this way, 10 trypsin-like proteins adjacent to annotated KLKs in *Xenopus tropicalis* were identified as putative KLK genes (XtKLKun1-10). Similarly, two putative KLKs were identified adjacent to annotated KLKs in *Anolis carolinensis* (AcKLK14L, AcKLKL), and three in *Gallus gallus* (GgKLK, GgKLKL, GgPRSS3), as shown in Figure 5 (also see Figure S2). Phylogenetically, all trypsin-like proteins identified in chicken, belong to the trypsin clade, and do not group with other kallikreins (Figure 4). Therefore, no true tissue kallikreins have yet been identified in chicken. The KLK-like proteins from the lizard, the turkey and the Zebra Finch do not group confidently within a specific KLK clade, but they do group with other tissue kallikreins (Figure 4). This supports an early origin of tissue kallikreins in aves and lepidosauria, as reported previously [22]. As for the amphibia, *Xenopus* contains orthologs of KLKB1, complement factor D (XtCFD), and trypsin (XtPRSS2). The other trypsin-like putative KLKs from *Xenopus* form two distinct groups (Figure 4). *Xenopus* trypsin-like Group1 proteins group with the trypsin clade, while *Xenopus* Group2 proteins group within the tissue kallikrein clade, but with only marginal statistical support. Therefore, it is possible that multiple, divergent tissue kallikreins also exist in the amphibia.

### Evolution of KLK Alternative Transcripts

**Figure 6:**
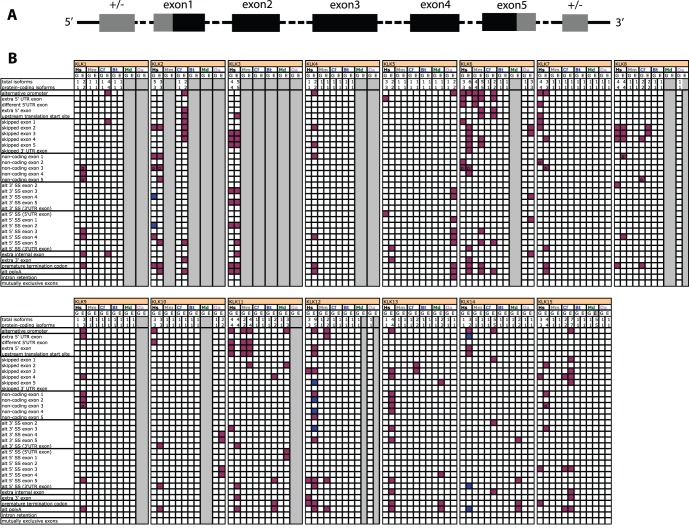
Features of the tissue KLK isoforms in human, mouse, dog, cattle, opossum and platypus. Panel A: The general structure of the reference KLK isoform includes 5 coding exons, and may include one 5′ and/or one 3′ UTR exon shown with a +/− sign [20]. Coding regions are shown in black, UTR in grey, dotted lines indicate that the length of introns may vary. Exon1 usually includes at least part of the 5′UTR, exon5 usually includes at least part of the 3′UTR. Panel B: Alternative splicing patterns for all annotated KLK isoforms in the NCBI Gene and Ensembl66 databases are shown. The different types of alternative splicing used to generate structural diversity (alternative promoter, exon skipping, alternative 3′ SS selection, alternative 5′ SS selection, alternative poly(A)) are as described previously [27]. Purple boxes indicate that a feature is used in a protein-coding isoform, blue boxes are for features only found in NMD or RNA-only isoforms. “p” indicates that only part of an exon has become non-coding. For each KLK, we chose as reference the isoform containing 5 coding exons, with or without one 5′ UTR and one 3′ UTR, to infer the most parsimonious diversity pattern (see panel A). Some of the Ensembl gene summaries, which form the basis of the data summarized in this panel, are shown in Figure S4, for human, mouse and dog KLKs, which display significant structural variability.

Phylogenetic reconstruction of the tissue and plasma kallikreins including all annotated protein-coding isoforms (KLKs-all dataset), shows that the alternative transcripts for each KLK form monophylletic groups (Figure S3). Moreover, isoforms from each species group closer to each other, than to isoforms of other species, indicating that alternative transcripts are species-specific. This is in agreement with the raw data from the databases, i.e. for example, apart from KLK4, KLK10, and KLK14, all other human KLKs have multiple protein isoforms (Table S1); however, in mouse, only Kl1b26, KLK6 and KLK11 have reported alternative isoforms, and in cattle, only KLK11 has reported alternative isoforms, while there are no alternative isoforms reported in hyrax and wallaby (Table S1). This is illustrated in more detail in Figure 6, where the different KLK isoforms are categorized based on their structural diversity and the different types of alternative splicing used to generate this diversity (alternative promoter, exon skipping, alternative 3′ SS selection, alternative 5′ SS selection, alternative poly(A) [27], also see Figure S4). Although certain isoform characteristics are shared for some kallikreins between species (e.g. KLK6 in human, mouse and dog; KLK8 in human and dog; KLK11 in human and mouse), there is no overall conservation of isoform types between species, or between the different kallikreins. Some alternative transcripts which have been reported previously, are not included in the databases used, for example KLK6 alternative transcripts in cattle, which use alternative promoters and differ in the 5′ UTR region [41]. Although this again indicates that similar structural diversity in the 5′UTR of KLK6 is shared between different species, the overall conclusion remains that, by and large, KLK isoform diversity seems to have evolved in a type-specific and species-specific manner. Splice variants for canine KLK9 and canine KLK14 have also been reported [42], [43], but again, these do not display the same type of structural diversity as the isoforms reported in human or other species. Although we have restricted our analysis to the annotated isoforms in the Refseq and Ensembl databases, which may not be complete, each database is consistent in the methods it uses to detect, filter, and annotate alternative transcripts (e.g. http://www.ensembl.org/info/docs/genebuild/ase_annotation.html). Therefore, despite any bias, we believe that the conclusions regarding isoform evolution within the kallikrein family, in any given species, are well-supported. Data between different species is difficult to compare, as the EST databases may not be of equal quality or depth of coverage, especially for the earlier diverging mammals. Nevertheless, for relatively closely related species, such as human and mouse, genome annotations are of equivalent quality.

The evolution of alternative transcripts in mammals has been studied in a number of gene families, showing that some are conserved across species and others are truly species-specific [26], [44], [45], [46], [47], [48], [49]. Two recent papers conclude that global alternative splicing patterns are species-specific [50], [51], and that the changes are often associated with the availability of splicing factor binding sites in introns. In addition, the observation of cancer-specific alternative transcripts in human diseased tissues argues for the potential to constantly evolve new isoforms [52], although cancer-specific isoforms have not been reported in other species [42], [43]. Our data indicate that, by and large, alternative splicing patterns are not conserved between species but develop independently in each species, at least for this gene family. In addition, in the tissue kallikrein family, which appears to have diverged via gene duplication coincident with the divergence of mammals, there is no conservation of isoform diversity between the different KLK types.

When only the human kallikreins are examined in more detail, alternative transcripts group into monophyletic groups, for all KLKs except KLK13 (Figure 3). The seven alternative transcripts of the plasma kallikrein consistently group together, with posterior probability of 1 in the Bayesian analysis (albeit with lower support by maximum likelihood methods). The tree also provides support for the following KLK subfamilies: KLK1, KLK2, and KLK3; KLK4 and KLK5; KLK9 and KLK11.

### Conclusions

This is the first study to specifically address the evolutionary relationships between tissue and plasma kallikreins. The two types of kallikreins belong to the same family of serine proteases [4] and show high sequence similarity in the region encoding the trypsin-like domain (>50%), as well as a similar exon/intron architecture, which might argue for a common origin. The comparative genomics and phylogenetic analysis argues for an earlier origin of plasma kallikrein than tissue kallikreins. Clear orthologs of plasma kallikrein are identified in non-mammalian species such as chicken, lizard, and frog, and form a well-supported monophyletic group. The plasma kallikreins also group closer to chymotrypsin and trypsin than to tissue kallikreins. In contrast, tissue kallikrein homologs from chicken, Zebra Finch, turkey, lizard, and frog are difficult to assign with confidence within the tissue kallikrein family. Moreover, the phylogeny indicates a rapid divergence of the tissue kallikreins in the mammals, with a more recent expansion of the “classical” kallikreins (KLK1, KLK2, and KLK3) in eutheria and primates. This is in agreement with the gene locus organization of the tissue kallikreins, which is very similar between human, mouse, dog, cattle, and opossum, as well as related species [22]. More complete genome assemblies for earlier diverging species, such as the hyrax, wallaby and platypus, may lend further support to this conclusion. The results of this study also provide support for certain subfamilies within the KLKs, at least one composed of KLK4 and KLK5, and another containing KLK9 and KLK11, as seen in previous analyses [22], [23].

Both tissue and plasma kallikreins have multiple protein-coding alternative transcripts, and alternative isoforms for each kallikrein type group together in phylogenetic analysis. Our data also indicate that, with respect to the kallikreins, alternative isoforms evolve in a species-specific manner.

Kallikreins are thought to exert their major physiological roles by acting as proteases. Notably, examination of sequence conservation indicates that many human KLK isoforms are lacking either one or more of the residues thought to constitute the catalytic triad of this group of proteases. This indicates that many alternative transcripts encode kallikreins which lack protease catalytic activity, so their expression may be a way for the organism to control the protease activity of kallikreins in different tissues; something similar has been suggested for the expression of protease-like molecules of the ADAMs family which lack catalytic activity, and which may act as antagonists of true proteases [53]. Alternatively, expression of KLK protein isoforms with no protease activity may indicate that KLKs have other functions, at least in human. Indeed, KLK10 has been reported to lack trypsin-like enzymatic activity in ovarian ascites fluid [54], although overexpression of KLK10 is associated with lessened aggressivity of ovarian cancer [55]. Also, it has been reported that the proteolytic activity of PSA is not necessary for its role in generating reactive oxygen species (ROS) in prostate cancer [56]; however, more recent reports indicate that the enzymatic activity of PSA is linked to inhibition of ROS scavenging [57] and to the growth of prostate tumors [58]. The function of alternative transcripts, especially those that encode truncated proteins, is still largely unknown, although it is possible that they mediate signaling either at the mRNA, or at the protein level. Recent studies have shown that species-specific alternative splicing patterns are associated with disordered protein regions which mostly reside on the protein surface and mediate protein-protein interactions [50]; furthermore, tissue-specific alternatively splicing often alters the availability of phosphorylation sites, pointing to a connection with signaling mediated by kinases [51]. Given the indication from the present dataset, that alternative splicing patterns are species-specific, this means that each organism has different constraints as to how many isoforms are expressed, and whether these retain enzymatic activity.

## Supporting Information

Figure S1 **Multiple sequence alignment of human kallikreins with other serine proteases.** All protein-coding alternative transcripts for each of the human tissue kallikreins, as well as plasma kallikrein, trypsin, chymotrypsin, plasminogen, and complement factor D were aligned using Muscle (also see Alignment S1). Conserved residues are highlighted in blue, and the consensus sequence logo is shown below the sequence for conserved regions (image generated with Jalview). Part of the alignment, corresponding to the trypsin-like protease domain for each sequence for the longest transcript for each gene is shown in Figure 2. Based on this alignment, alternative transcripts that do not retain the catalytic triad residues were classified as lacking protease activity, as shown in Table S1.
(TIF)

Figure S2 **KLK gene clusters in selected species, as determined by examining the NCBI Gene database.** The genetic locus neighborhood for *KLK*s in human, mouse, dog, cattle, opossum, chicken, lizard, and frog are shown. The *KLK* gene clusters in human, mouse, dog and cattle have a similar structure, except that dog is missing KLK3, cattle is missing KLK2 and KLK3, and mouse is missing KLK2 and KLK3 and has an insertion, between KLK1 and KLK15, of 13 KLK-like genes as well as 10 KLK pseudogenes. The *KLK* gene cluster in the opossum, lizard, frog, and chicken are as described in Figure 5. Note that the lizard and frog sequences are on parts of the genome that are not fully assembled yet.
(EPS)

Figure S3 **Phylogenetic reconstruction of all identified homologs of plasma and tissue kallikreins, from the 13 species studied, including all annotated protein-coding alternative transcripts.** The tree shown is based on maximum likelihood analysis using PhyML. Similar results were obtained with RaxML, while MrBayes analysis with 3 million generations failed to converge on a tree. Numerical values at the nodes of the tree indicate statistical support based on 100 bootstraps. Full details and accession numbers for all protein sequences used are given in Table S1; also see Alignments S5 and S6, for the masked and trimmed alignments used to construct the tree.
(EPS)

Figure S4 **Ensembl gene summaries showing the exon/intron boundaries for human, mouse and dog KLKs, which display significant structural variability.** These transcript views form the basis of the data summarized in Figure 6. For all protein-coding isoforms shown, the numbers to the right indicate the order of coding exons, missed numbers indicate skipped exons, ‘s’ denotes a shorter exon generated by alternative 3′ or 5′ splice site choice, “or” next to an exon number denote different exons, while ‘in’ denotes extra internal exons. No clear pattern of shared structural variability is seen when comparing different KLKs, or when comparing the same KLK from different species.
(TIF)

Table S1 **Accession numbers of all sequences used in this study.** Refseq and ENSEMBL accession numbers are given for all annotated protein-coding transcripts of the sequences mentioned in the text. The gene name column lists the abbreviations used in the phylogenetic table and the multiple alignments. Transcript and exon supporting evidence from ENSEMBL is indicated, where available. For all human kallikrein sequences, column M lists the presence (Y) or absence (N) of the important residues of the catalytic triad [18], highlighted in Figure 2: H - His41, D - Asp96, S - Ser189 (trypsin numbering).
(XLS)

Alignment S1 **Masked alignment for the KLKs-all_human dataset.** For each dataset, the alignment was produced using the program Muscle. Manual masking and trimming was performed in McClade. The files were then exported in Phylip3.6 simple text format. The accession numbers for all sequences can be found in Table S1, using the sequence name abbreviation given in the beginning of each line. For each dataset, the “masked” alignment includes all full length sequences; the last line (which contains only ‘I’ characters and ‘−’ for gaps) was used to mark with an ‘I’ all unambiguous homologous residues retained for phylogenetic analysis. The trimmed alignment is the result of deleting all positions that are not marked with an ‘I’ in the masked alignment; the trimmed alignments were used for the phylogenetic analysis.
(PHY)

Alignment S2 **Trimmed alignment for the KLKs-all_human dataset; the KLKs-all_human dataset was used to construct the tree shown in** **Figure 3** **.** For each dataset, the alignment was produced using the program Muscle. Manual masking and trimming was performed in McClade. The files were then exported in Phylip3.6 simple text format. The accession numbers for all sequences can be found in Table S1, using the sequence name abbreviation given in the beginning of each line. For each dataset, the “masked” alignment includes all full length sequences; the last line (which contains only ‘I’ characters and ‘−’ for gaps) was used to mark with an ‘I’ all unambiguous homologous residues retained for phylogenetic analysis. The trimmed alignment is the result of deleting all positions that are not marked with an ‘I’ in the masked alignment; the trimmed alignments were used for the phylogenetic analysis.
(PHY)

Alignment S3 **Masked alignment for the KLKs-one dataset.** For each dataset, the alignment was produced using the program Muscle. Manual masking and trimming was performed in McClade. The files were then exported in Phylip3.6 simple text format. The accession numbers for all sequences can be found in Table S1, using the sequence name abbreviation given in the beginning of each line. For each dataset, the “masked” alignment includes all full length sequences; the last line (which contains only ‘I’ characters and ‘−’ for gaps) was used to mark with an ‘I’ all unambiguous homologous residues retained for phylogenetic analysis. The trimmed alignment is the result of deleting all positions that are not marked with an ‘I’ in the masked alignment; the trimmed alignments were used for the phylogenetic analysis.
(PHY)

Alignment S4 **Trimmed alignment for the KLKs-one dataset; the KLKs-one dataset was used to construct the tree shown in** **Figure 4** **. For each dataset, the alignment was produced using the program Muscle.** Manual masking and trimming was performed in McClade. The files were then exported in Phylip3.6 simple text format. The accession numbers for all sequences can be found in Table S1, using the sequence name abbreviation given in the beginning of each line. For each dataset, the “masked” alignment includes all full length sequences; the last line (which contains only ‘I’ characters and ‘−’ for gaps) was used to mark with an ‘I’ all unambiguous homologous residues retained for phylogenetic analysis. The trimmed alignment is the result of deleting all positions that are not marked with an ‘I’ in the masked alignment; the trimmed alignments were used for the phylogenetic analysis.
(PHY)

Alignment S5 **Masked alignment for the KLKs-all dataset.** For each dataset, the alignment was produced using the program Muscle. Manual masking and trimming was performed in McClade. The files were then exported in Phylip3.6 simple text format. The accession numbers for all sequences can be found in Table S1, using the sequence name abbreviation given in the beginning of each line. For each dataset, the “masked” alignment includes all full length sequences; the last line (which contains only ‘I’ characters and ‘−’ for gaps) was used to mark with an ‘I’ all unambiguous homologous residues retained for phylogenetic analysis. The trimmed alignment is the result of deleting all positions that are not marked with an ‘I’ in the masked alignment; the trimmed alignments were used for the phylogenetic analysis.
(PHY)

Alignment S6 **Trimmed alignment for the KLKs-all dataset; the KLKs-all dataset was used to construct the tree shown in Figure S3.** For each dataset, the alignment was produced using the program Muscle. Manual masking and trimming was performed in McClade. The files were then exported in Phylip3.6 simple text format. The accession numbers for all sequences can be found in Table S1, using the sequence name abbreviation given in the beginning of each line. For each dataset, the “masked” alignment includes all full length sequences; the last line (which contains only ‘I’ characters and ‘−’ for gaps) was used to mark with an ‘I’ all unambiguous homologous residues retained for phylogenetic analysis. The trimmed alignment is the result of deleting all positions that are not marked with an ‘I’ in the masked alignment; the trimmed alignments were used for the phylogenetic analysis.
(PHY)
