# Supplementary figures and images for: Evolution of the Plasma and Tissue Kallikreins, and Their Alternative Splicing Isoforms
Source: PLoS One. 2013 Jul 10;8(7):e68074. doi: 10.1371/journal.pone.0068074 (PMC3707919; doi:10.1371/journal.pone.0068074)

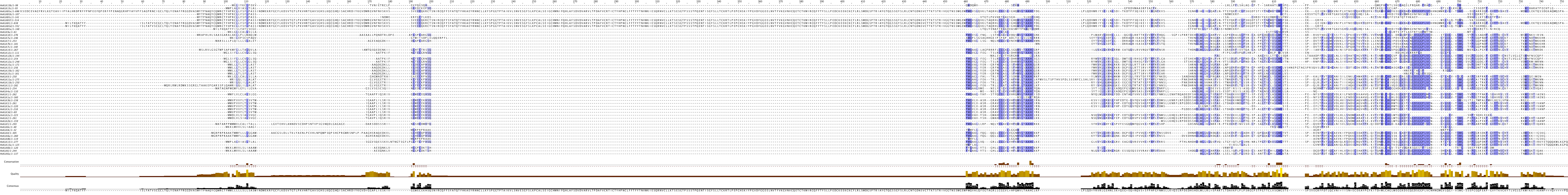

Supplement: Figure S1 — Multiple sequence alignment of human kallikreins with other serine proteases. All protein-coding alternative transcripts for each of the human tissue kallikreins, as well as plasma kallikrein, trypsin, chymotrypsin, plasminogen, and complement factor D were aligned using Muscle (also see Alignment S1). Conserved residues are highlighted in blue, and the consensus sequence logo is shown below the sequence for conserved regions (image generated with Jalview). Part of the alignment, corresponding to the trypsin-like protease domain for each sequence for the longest transcript for each gene is shown in Figure 2. Based on this alignment, alternative transcripts that do not retain the catalytic triad residues were classified as lacking protease activity, as shown in Table S1. (TIF) [file pone.0068074.s001.tif]

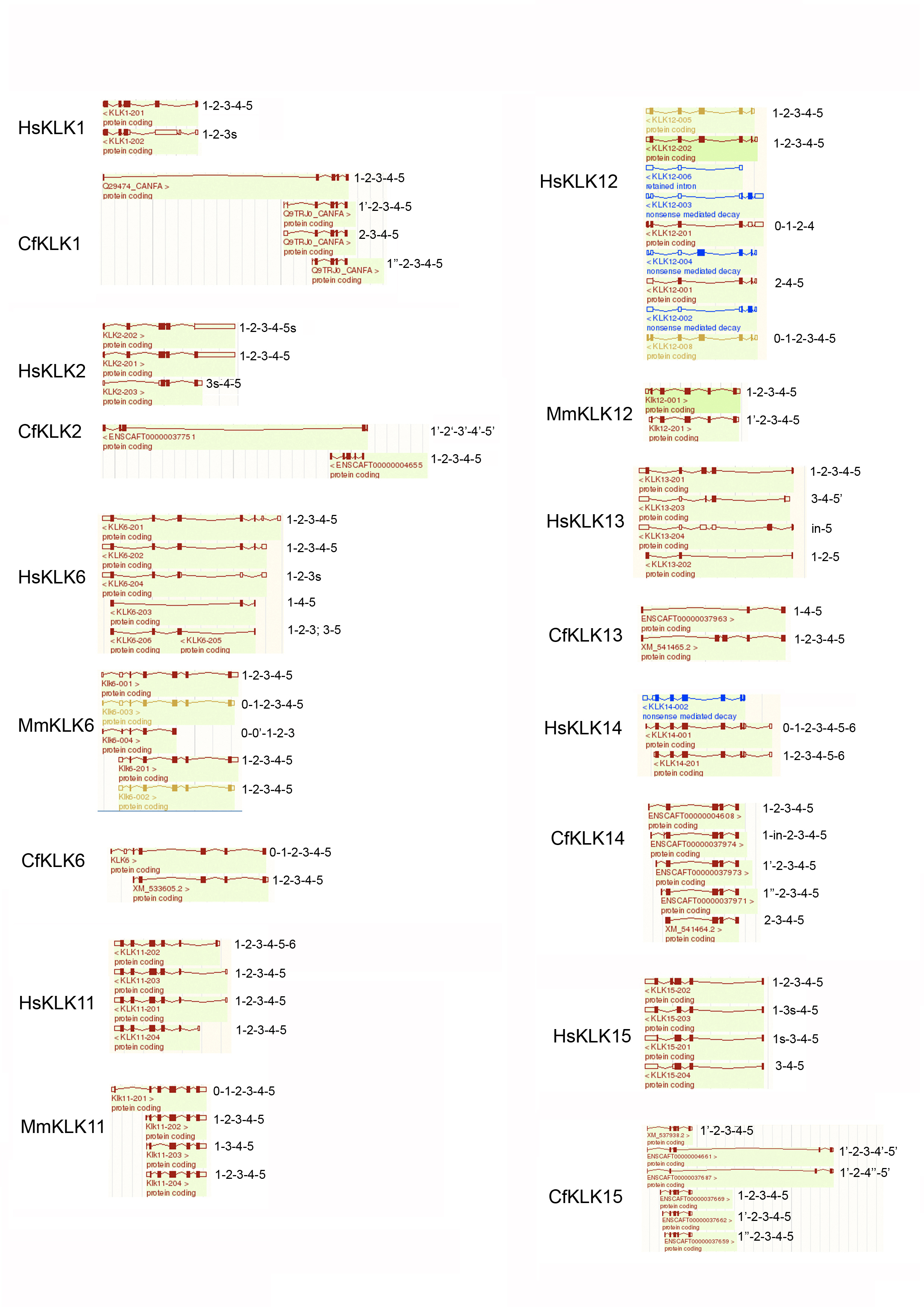

Supplement: Figure S4 — Ensembl gene summaries showing the exon/intron boundaries for human, mouse and dog KLKs, which display significant structural variability. These transcript views form the basis of the data summarized in Figure 6. For all protein-coding isoforms shown, the numbers to the right indicate the order of coding exons, missed numbers indicate skipped exons, ‘s’ denotes a shorter exon generated by alternative 3′ or 5′ splice site choice, “or” next to an exon number denote different exons, while ‘in’ denotes extra internal exons. No clear pattern of shared structural variability is seen when comparing different KLKs, or when comparing the same KLK from different species. (TIF) [file pone.0068074.s004.tif]
